# Discharge From the Acute Hospital Setting on Postoperative Day One Following Selective Dorsal Rhizotomy: An Illustrative Pediatric Case and Literature Review

**DOI:** 10.7759/cureus.92695

**Published:** 2025-09-19

**Authors:** Lisa B Shields, Ian S Mutchnick

**Affiliations:** 1 Neurological Surgery, Norton Neuroscience Institute, Norton Healthcare, Louisville, USA; 2 Neurosurgery, Norton Children’s Hospital and Norton Children's Neuroscience Institute, Norton Healthcare, Louisville, USA; 3 Neurosurgery, University of Louisville, Louisville, USA

**Keywords:** hospital length of stay, pediatric neurosurgery, pediatric surgery, rehabilitation, selective dorsal rhizotomy, titanium clips

## Abstract

A selective dorsal rhizotomy (SDR) is a neurosurgical procedure aimed at improving lower extremity spasticity in children. The traditional postoperative course involves strict bed rest for 24-48 hours and an acute hospital length of stay usually ranging between three and five days. We present the case of a seven-year-old male with cerebral palsy and right-sided spasticity secondary to a perinatal ischemic infarction in the left hemisphere. The patient underwent a right-sided SDR consisting of a one-level laminectomy at the conus medullaris. The medium-sized Anastoclip GC Closure System was used to close the dura. The patient was admitted to the intensive care unit postoperatively for one night and was not required to lie flat postoperatively. He was out of bed on postoperative day (POD) zero and engaged in physical therapy on POD one. This patient is the first post-SDR reported to be discharged from the acute hospital setting on POD one to inpatient rehabilitation. The patient was able to stand flat-footed bilaterally within four weeks of the SDR, although he reported continued balance issues with running and jumping. This case illustrates the potential to minimize the postoperative stay of SDR patients safely using Anastoclips, repleting the cerebrospinal fluid volume after rhizotomy with lactated Ringer’s, and eliminating the postoperative bed rest.

## Introduction

A selective dorsal rhizotomy (SDR) is a neurosurgical technique designed to improve lower extremity spasticity, gain muscle strength, enhance gait speed, and improve gross motor function in patients with spastic cerebral palsy [1-3]. The operative procedure differs based on the level of exposure, extent of laminectomy, and identification of nerve roots [2]. The surgery may consist of a multi- or single-level laminectomy or a keyhole interlaminar method [4-6].

Complications may ensue following this procedure, including headaches, nausea, and cerebrospinal fluid (CSF) leaks [7,8]. With an incidence ranging between 2.7% and 7.1% following intradural spinal surgery in the pediatric population, CSF leaks are also associated with several potential complications [5,6,9,10]. To reduce the CSF leak complication rate, patients often lie flat postoperatively for 24-48 hours [11-13]. The acute hospital length of stay (LOS) after an SDR usually ranges between three and five days [12-15]. A shorter LOS following SDR is valuable for several reasons, including decreased risk of complications, lower costs, and a more favorable experience for patients and their families. It has been reported that physical therapy (PT) may not initiate until day three or four after an SDR [12].

Here, we present the first reported case of a patient with cerebral palsy and right-sided spasticity secondary to a perinatal ischemic infarction in the left hemisphere who was discharged from an acute hospital on postoperative day (POD) one following an SDR. The purpose of our study was to determine whether a pediatric patient who undergoes an SDR can be discharged from the acute hospital setting on POD one and not experience any subsequent adverse effects. Factors for this expedited discharge are discussed, including the importance of repleting the volume of lost CSF intraoperatively to mitigate postoperative nausea and vomiting and mobilizing patients out of bed as early as POD zero.

## Case presentation

History and radiological findings

An 8 lb 2.9 oz (3,710 g) male was born at 39 weeks and four days of gestation by cesarean section. His Apgar score was 5 out of 7 at birth, and he was intubated due to hypoxia and spent several hours in the neonatal intensive care unit. The patient was diagnosed with cerebral palsy and right-sided spasticity secondary to a perinatal ischemic infarction in the left hemisphere. All genetic studies were normal. The patient underwent botulinum toxin injections to the right upper and lower extremities on three occasions when he was two years old and to the right medial hamstring and gastrocnemius muscles twice two years later.

The patient was evaluated by a pediatric neurosurgeon at age six years. On examination, the right upper and lower extremities were hypertonic, and the right lower extremity was hyperreflexic. Right toe walking was observed. The patient wore an ankle-foot orthosis for his right lower extremity at night. He was an independent ambulator with an asymmetric pattern with a toe-heel gait on the right with a whipping motion from the spasticity in the tibialis posterior muscle. Running increased his asymmetry with movement. He was independent on level surfaces and stairs, but had difficulty descending stairs using a reciprocal pattern. His neurological examination revealed responsive right upper extremity tone with proprioceptive loading. The Modified Ashworth Scale is presented in Table 1. The Gross Motor Function Classification System (GMFCS) four months preoperatively was II, indicating the ability to walk with limitations, sometimes using a handrail, and meeting the feet on the same step.

**Table 1:** Modified Ashworth Scale.

Muscles	Right	Left
Rectus femoris	0	0
Hamstrings	2	0
Adductor longus	2	0
Tibialis anterior	0	0
Gastrocnemius	2	0

**Figure 1:**
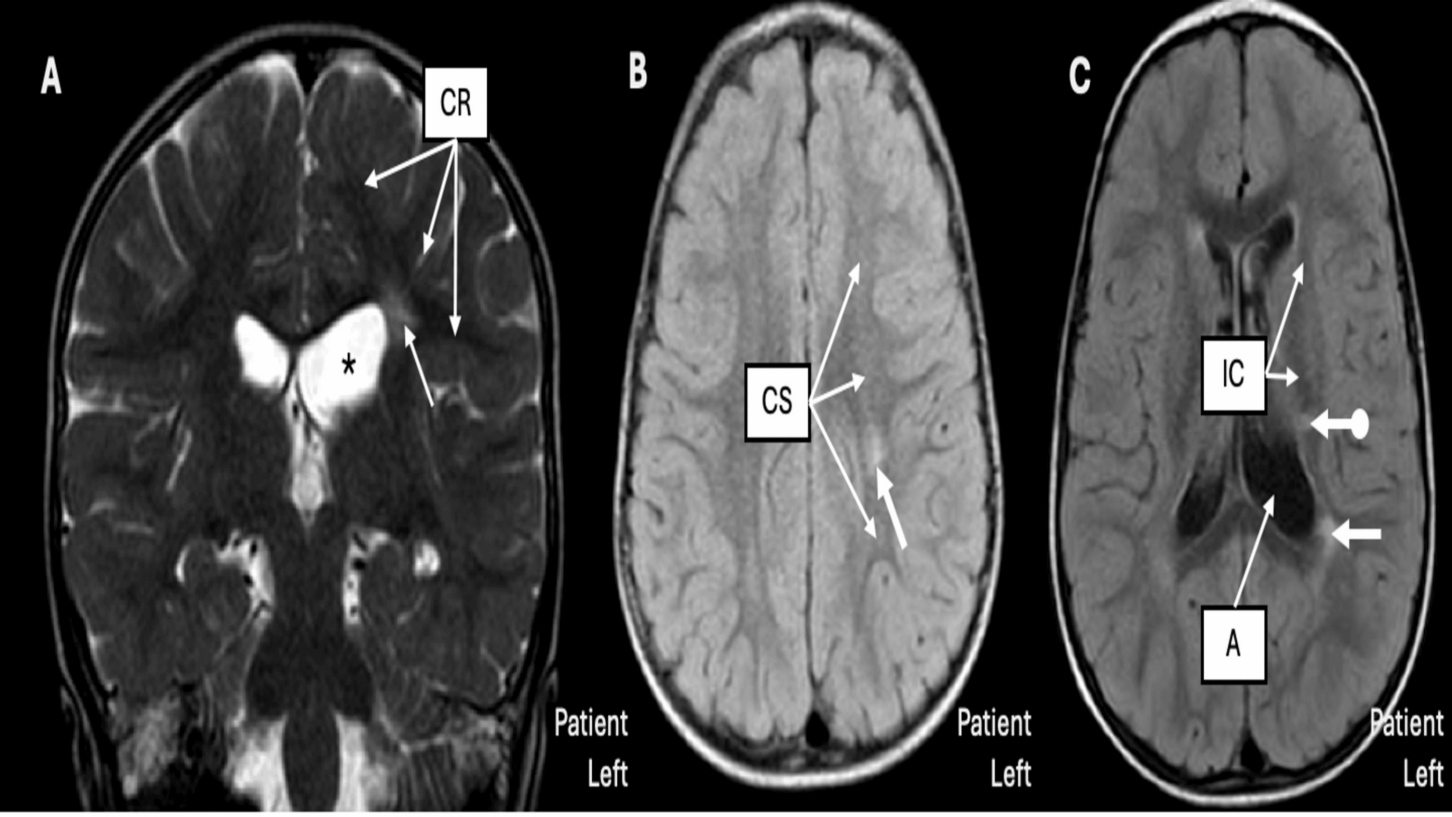
Brain MRI findings. Brain MRI demonstrating locations of the periventricular leukomalacia – radiographic evidence of early white matter damage. (A) Coronal T2 MRI showing the left corona radiata (CR), with the thick white arrow demonstrating the leukomalacia. Note the enlarged ventricle (*), confirming white matter loss in that region. (B) Axial fluid-attenuated inversion recovery showing the left centrum semiovale (CS) with the thick arrow showing the location of the leukomalacia. (C) Axial T1 showing both the internal capsule (IC) and ventricular atrium (A) with the periatrial leukomalacia (thick arrow) and the leukomalacia of the internal capsule (thick arrow with the ball tip).

**Figure 2:**
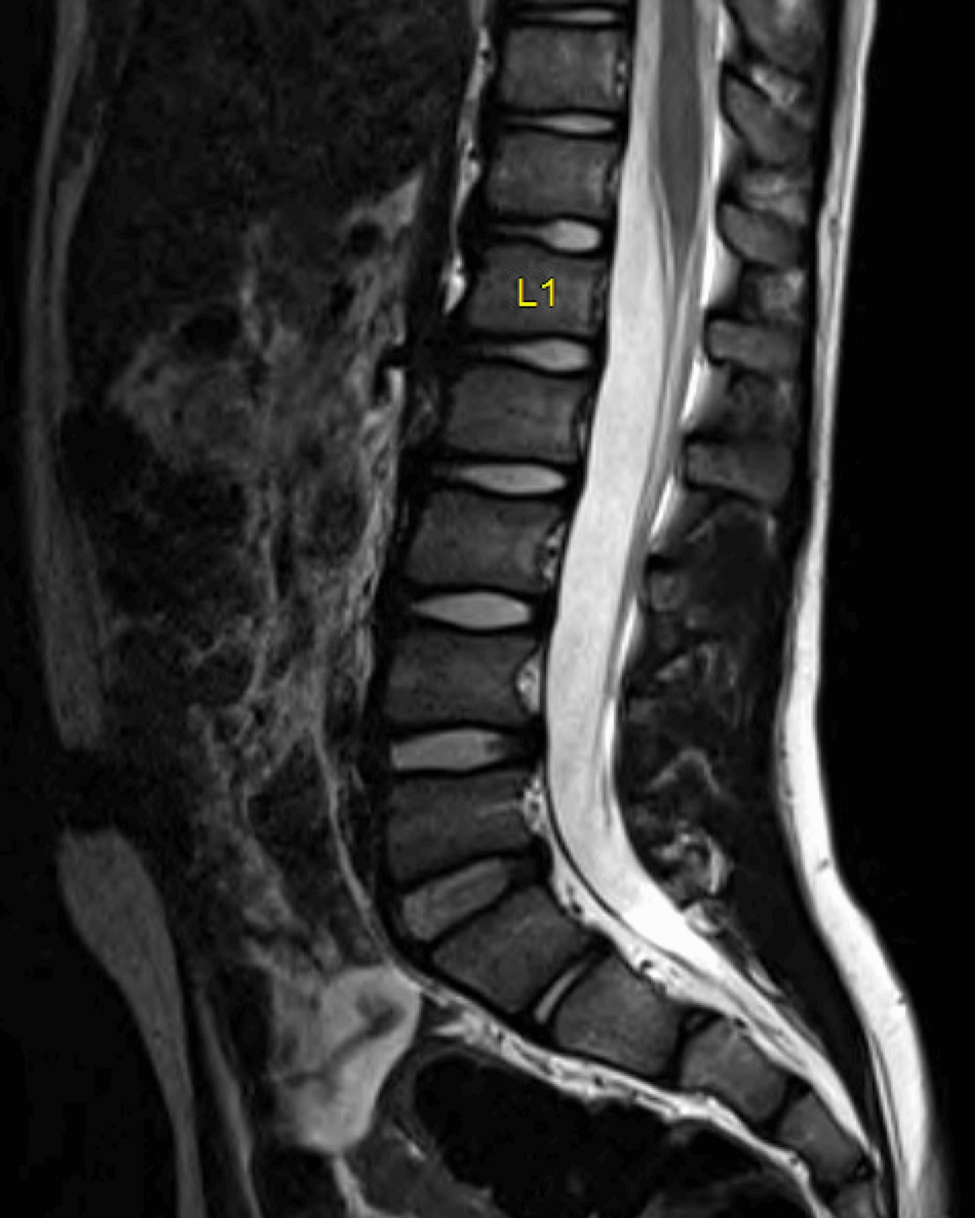
Lumbar MRI findings. A sagittal lumbar T2 MRI without abnormalities. The conus medullaris terminates at the level of the mid-body of L1, allowing adequate localization of the single-level laminectomy required to access the cauda equina allowing for access to the lumbar nerve roots.

During the patient’s appointment with pediatric rehabilitation preoperatively, there was 3 out of 5 strength of right dorsiflexion, increased tone in the right ankle, and no ankle clonus. The patient was hemiplegic, although he was able to place his right heel on the ground. The Galleazzi test was negative. Table 2 describes the preoperative characteristics, perioperative course, and postoperative rehabilitation milestones. A brain MRI demonstrated chronic white matter involving the periatrial white matter, left centrum semiovale, corona radiata, and internal capsule (Figure 1). A lumbar MRI was unremarkable, with the conus medullaris terminating at the level of the mid-body of L1 (Figure 2).

**Table 2:** Preoperative characteristics, perioperative course, and postoperative rehabilitation milestones. POD: postoperative day; ICU: intensive care unit; PT: physical therapy; GMFCS: Gross Motor Function Classification System

Preoperative characteristics	Perioperative course	Postoperative rehabilitation milestones
3/5 strength of right dorsiflexion	Underwent one-level laminectomy with dural closure using AnastoClip GC Closure System	Right lower extremity: 4/5 hip and knee flexion, 2/5 ankle plantar flexion and dorsiflexion
Increased tone in the right ankle	Admitted to the post-anesthesia care unit postoperatively for one night	Tight right Achilles tendon and hamstring
No ankle clonus	Not required to lie flat after the surgery	Patient-Specific Functional Scale: 11
Hemiplegic but able to place the right heel on the ground	Out of bed on POD zero in the ICU	Four weeks postoperatively: stand flat-footed bilaterally; continued balance issues with running and jumping
Galleazzi test negative	PT on POD one	Four months postoperatively: 4+/5 right ankle plantar flexion and dorsiflexion; ambulated independently with no assist device; mild steppage gait with hip hiking and genu valgum on the right; no spasticity or clonus
Hypertonic upper and lower extremities	Discharged from the acute hospital setting on POD one	
Hyperreflexic right lower extremity		
Right toe walking		
Modified Ashworth Scale (Table 1)		
GMFCS four months preoperatively: II		
Toe-heel gait on the right with whipping motion from spasticity in the tibialis posterior muscle		
Running increases asymmetry with movement		
Difficulty descending stairs using a reciprocal pattern		

Selective dorsal rhizotomy

**Figure 3:**
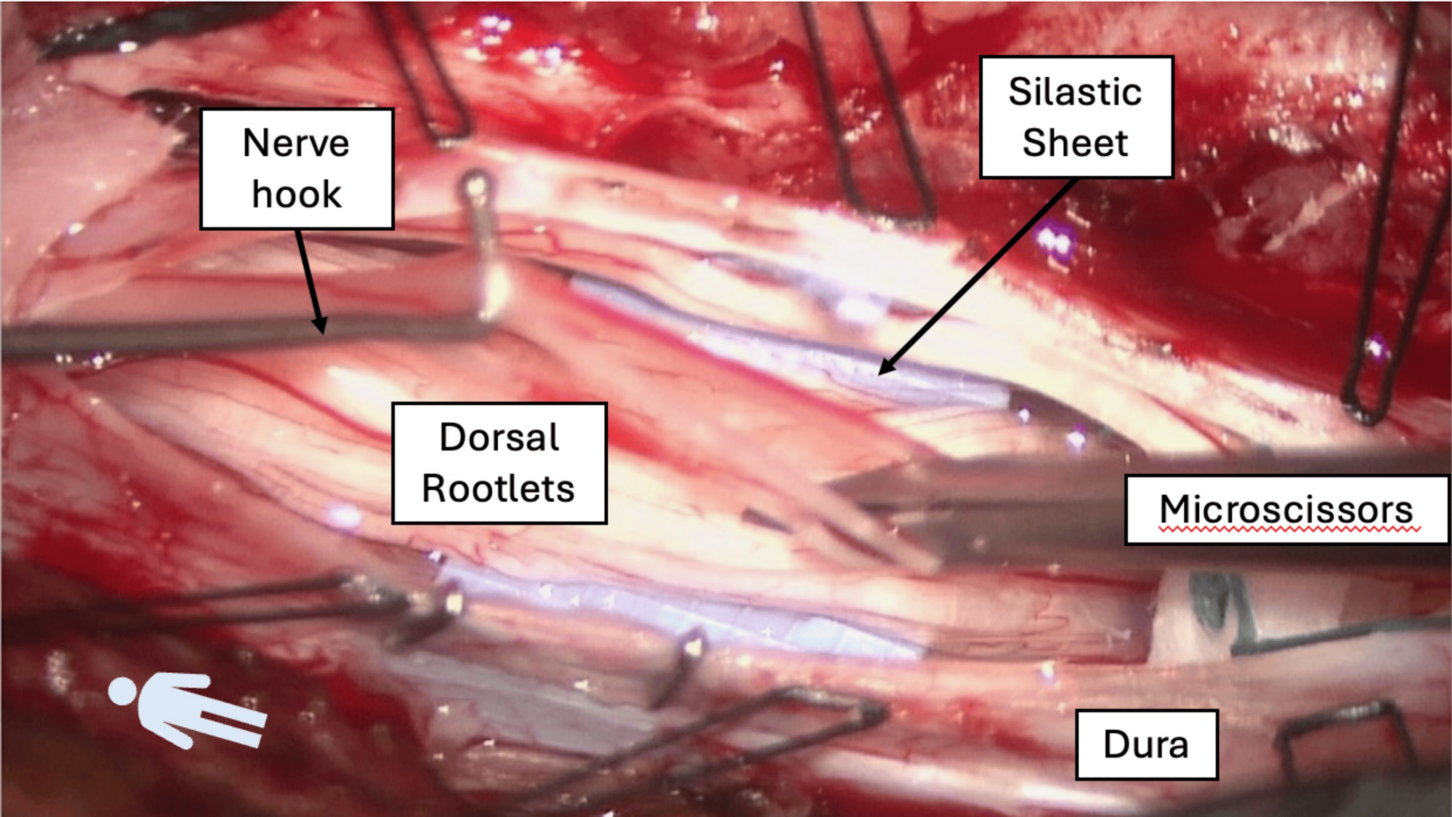
Operative field in a selective dorsal rhizotomy. The lamina of L1 has been removed, and the dura has been opened. The dorsal nerve rootlets have been separated from the ventral rootlets by the silastic sheet. Using a nerve hook and microscissors, the rootlets are fasciculated and stimulated (not shown) to determine if a particular rootlet needs to be cut – the basis of the “selective” dorsal rhizotomy.

The patient underwent an SDR consisting of a one-level laminectomy with dural closure using the AnastoClip GC Closure System [16]. Following removal of the lamina of L1, the ligamentum flavum and epidural fat were harvested en bloc. The dura was opened, and the arachnoid membrane was microdissected. The filum was isolated and severed after confirming by stimulation testing that it lacked neural function. Each dorsal nerve root was isolated and fasciculated, and rhizotomies were performed based on neurophysiological interrogation findings (Figure 3). Only right-sided rootlets were cut in this case, though we routinely test both sides with unilateral spasticity. Anastoclips were used to partially close the dura. A volume of 30 mL of body temperature lactated Ringer’s solution was instilled with an angiocatheter on a 30 mL syringe. The durotomy was fully closed with Anastoclips, and the ligamentum flavum and epidural fat from the L1 laminectomy en bloc were placed over the durotomy. Surgicel covered the autologous tissue graft.

Postoperative course

The patient was admitted to the post-anesthesia care unit postoperatively for one night. The patient was not required to lie flat after the surgery, and he was out of bed on POD zero in the intensive care unit. He underwent PT on POD one and was discharged from the acute hospital setting on the same day. The patient then spent 14 days in an inpatient rehabilitation program. Upon discharge from the inpatient rehabilitation program, the following physical examination findings of the patient’s right lower extremity were observed: 4 out of 5 hip and knee flexion, and 2 out of 5 ankle plantar flexion and dorsiflexion. No spasticity was noted, although there was evidence of a tight right Achilles tendon and hamstring. The patient scored an 11 on the Patient-Specific Functional Scale.

At the patient’s neurosurgical appointment four weeks postoperatively, he was able to stand flat-footed bilaterally, although he reported continued balance issues with running and jumping. He manifested no clinical issues indicative of a CSF leak. At the patient’s last follow-up with pediatric rehabilitation four months postoperatively, there was 4+ out of 5 right ankle plantar flexion and dorsiflexion. The patient ambulated independently with no assist device. A mild steppage gait was observed with hip hiking and genu valgum on the right. There was no spasticity and no clonus.

## Discussion

Complications may arise after an SDR, including headaches, nausea, and CSF leaks (Table 3) [7,8]. CSF leaks are also associated with myriad complications (Table 3) [5,6,9,10].

**Table 3:** Potential complications following a selective dorsal rhizotomy. CSF: cerebrospinal fluid

Complication
Nausea
Vomiting
Prolonged wound healing
Superficial wound infections
Urinary retention
Headaches
CSF leak
Positional headaches
CSF fistulas
Meningitis
Arachnoiditis
Intracranial hypotension
Nerve root impingement
Pseudomeningoceles
Wound infections

The acute hospital LOS following an SDR usually ranges between three and five days, often with a period of mandatory bed rest lasting 24-48 hours in the hopes that this will reduce the risk of CSF leakage [12-15]. Most importantly, an SDR involves a single-level laminectomy. The amount of bony exposure required for an SDR varies from a single-level laminectomy to a multi-level laminoplasty [1]. Because neurophysiological interrogation techniques are not yet standardized and not yet definitively validated to provide the material information required for a selection process, many surgeons worldwide perform multi-level exposures so that nerve root levels can be verified and appropriate percentages of those roots can be sectioned. We believe that our interrogation technique is robust enough that root-level verification is not necessary. Our technique appears to provide excellent identification of nerve rootlets that need sectioning without requiring knowledge of which foramen the particular rootlet is exiting. This allows us to perform a one-level laminectomy, which is a key part of our attempt to reduce postoperative LOS. Ou and colleagues reported that the hospital LOS was significantly decreased after a single-level versus a multi-level laminectomy (3.4 vs. 5.2 days, respectively; p = 0.01) [15].

Second, a large volume of CSF is lost during an SDR as the operative field must continually remain clear; CSF is suctioned out continually during the operation. We opine that approximately only one-third of the normal CSF volume is present in the neuraxis after an SDR, though there is no clear data to verify this. This depletion of CSF may cause severe headaches, nausea, and emesis for up to 48 hours postoperatively, delaying mobilization and discharge. To attempt to compensate for this and with a mind toward minimizing the postoperative recovery time, we inject a volume of lactated Ringer’s solution (which more closely resembles CSF than normal saline) following an SDR. It is hoped that the postoperative morbidity is mitigated by repleting the CSF volume. This may also account for the shortened acute hospital LOS following an SDR, though a focused study would be necessary to demonstrate this empirically. The minimal and low-risk effort required to place 30-45 mL of body temperature lactated Ringer’s intradurally just before complete closure of the durotomy takes only about three minutes and has not imposed a safety risk [14].

Third, a watertight and reliable closure is crucial. In our recent study of Anastoclips among 297 consecutive patients, only four had clinical evidence of a CSF leak, and none required an operative repair [14]. A total of 128 of these patients had no bed rest restrictions and were mobilized on POD zero. We assert that Anastoclips provide a reliable and durable watertight closure that appears to withstand very early postoperative mobilization, including a discharge to inpatient rehabilitation on POD one after a rhizotomy in this case.

Fourth, our technique for closure includes replacement of both the native epidural fat, as well as the ligamentum flavum, which may have implications for healing [14]. Epidural fat is a unique adipose tissue with less and thinner connective tissue than subcutaneous fat, which most surgeons consider to be an indispensable part of the approach in spinal surgeries [17-23]. However, several studies beginning in the 1990s have found that pathological or iatrogenic reduction of epidural fat is an important source of dural adhesion, scar/fibrosis formation, and failed back surgery syndrome [17,19,21,22]. The emerging research on the cross-talk between epidural fat and dura mater deserves our attention in this context of a dorsal iatrogenic durotomy. Al-Jezani et al., at the McCaig Institute for Bone and Joint Health at the University of Calgary, have reported the isolation of stem cells from human epidural fat [24]. Shah subsequently reported a series of murine experiments that demonstrated an expansion of stem cell populations during the growth or maturation period, a reduction in stem cell numbers when these animals reached skeletal maturity, and a loss of α-smooth muscle actin expression in the dura when stem cell populations were ablated (the actin is a biomarker for dural tissue integrity) [23]. Crucially, they also reported that there was a clear increase in stem cells at sites of dural injury. These authors hypothesized that the stem cells are native to epidural fat (and not the dura) as assay markers for stem cells remained consistent in the epidural fat over the time points examined, whereas there was an expansion followed by a reduction of this population over time in the dura mater following injury. These authors concluded that “epidural fat and/or dural [stem cells] contribute to the homeostasis of dural tissue over the course of normal growth and maturation, and that these [stem cells] are involved in tissue repair post-injury” [23]. This hypothesis will require more research to determine, but there is solid preliminary evidence that epidural fat may facilitate dural repair following surgical durotomy and closure.

While not all SDR patients will be candidates for a POD one discharge to rehabilitation, we feel this “proof of concept” can be built out to significantly reduce the LOS for these patients. One of the main barriers to this kind of efficient patient flow is the insurance approval for inpatient rehabilitation. To streamline this process, we have developed a team, including the pediatric physical medicine and rehabilitation physician, the pediatric liaison officer for the pediatric rehabilitation facility, and the medical assistant for the pediatric neurosurgery office. All are notified when an SDR is scheduled, and language aimed at facilitating insurance approval is incorporated into the consent at the last pediatric neurosurgical office visit before the surgery. Finally, postoperative PT and OT evaluations are expedited to meet insurance documentation requirements.

Strengths and limitations

The strength of this case report is that it features the first patient who underwent an SDR and was discharged from the acute hospital setting on POD one to inpatient rehabilitation. This case also highlights the ability to minimize the postoperative stay of SDR patients safely using Anastoclips, repleting the CSF volume following a rhizotomy with lactated Ringer’s solution, and eliminating the need for postoperative bed rest. The removal of the requirement of lying flat postoperatively was implemented at our Institution after no CSF leaks occurred when patients did not lie flat after surgery. While CSF repletion, watertight closure, and early mobilization facilitated earlier discharge, we acknowledge that this early discharge may be due to other possible contributors, such as patient selection, single-level exposure, and institutional rehabilitation planning. The limitation of this work is that it is a single-case design with a limited follow-up and a lack of generalizability. Additionally, this case is hypothesis-generating rather than practice-changing. Another limitation is that it lacks comprehensive standardized objective outcome measures at baseline and postoperatively, which limits its reproducibility.

## Conclusions

SDR is an effective procedure to treat patients with spastic diplegia. This case is the first post-SDR patient reported to be discharged from the acute hospital setting on POD one to inpatient rehabilitation. The patient was able to stand flat-footed bilaterally within four weeks of the SDR. By repleting the CSF volume intraoperatively, closing with Anastoclips, mobilizing the patients on POD zero, and starting PT on POD one, patients may be discharged from the acute hospital on POD one after an SDR. This advancement may be encouraging for patients and their families and may have positive financial implications for the healthcare system. The positive outcome in the current patient who underwent an SDR and was discharged from the acute hospital setting on POD one is a precursor for future randomized studies with a large sample size for meaningful evaluation.
